# Horizontal gene transfer converts non-toxigenic *Clostridium difficile* strains into toxin producers

**DOI:** 10.1038/ncomms3601

**Published:** 2013-10-17

**Authors:** Michael S.M. Brouwer, Adam P. Roberts, Haitham Hussain, Rachel J. Williams, Elaine Allan, Peter Mullany

**Affiliations:** 1 Department of Microbial Diseases, UCL Eastman Dental Institute, University College London, 256 Gray’s Inn Road, London WC1X 8LD, UK

## Abstract

*Clostridium difficile* is a major nosocomial pathogen and the main causative agent of antibiotic-associated diarrhoea. The organism produces two potent toxins, A and B, which are its major virulence factors. These are chromosomally encoded on a region termed the pathogenicity locus (PaLoc), which also contains regulatory genes, and is absent in non-toxigenic strains. Here we show that the PaLoc can be transferred from the toxin-producing strain, 630Δ*erm*, to three non-toxigenic strains of different ribotypes. One of the transconjugants is shown by cytotoxicity assay to produce toxin B at a similar level to the donor strain, demonstrating that a toxigenic *C. difficile* strain is capable of converting a non-toxigenic strain to a toxin producer by horizontal gene transfer. This has implications for the treatment of *C. difficile* infections, as non-toxigenic strains are being tested as treatments in clinical trials.

## Abstract (web summary)

*Clostridium difficile* produces potent toxins that are encoded by its pathogenicity locus. Here, Brouwer *et al.* demonstrate surprising bacterial genome plasticity whereby the pathogenicity locus is transferred from toxigenic to non-toxigenic strains of *C. difficile* by conjugational transfer.

## Graphical abstract

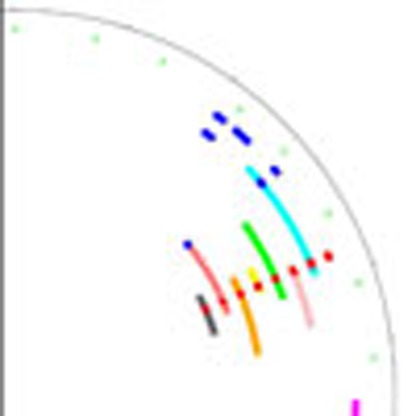

C*lostridium* *difficile*-associated diarrhoea (CDAD) can range from mild to severe and can be fatal due to complications such as pseudomembranous colitis and toxic megacolon^1^. CDAD is almost always associated with antibiotic usage, which disrupts the normal gut microflora allowing *C. difficile* colonization^2^. The organism produces two potent toxins, A and B, which disrupt the gut epithelium and are the major virulence factors of this organism^3^,^4^. Both toxins are encoded on the pathogenicity locus (PaLoc), a 19.6-kb chromosomal region that also encodes potential transcriptional regulators and a holin-like protein. The entire PaLoc is absent from non-toxigenic strains replaced by a highly conserved 115 bp non-coding region at this genomic site^5^.

*C. difficile* 630Δ*erm*^6^ is an erythromycin-sensitive derivative of the toxigenic *C. difficile* 630 (ref. 7) and contains the PaLoc^3^,^4^. This strain also contains at least six active conjugative transposons (CTns), genetic elements that are generally integrated into the genome but are capable of mediating their own transfer to suitable recipients^8^. Conjugative transfer was shown for CTn*1,* CTn*2*, CTn*4*, CTn*5*, CTn*7* and Tn*5397*, all of which can be transferred to the non-toxigenic *C. difficile* strain CD37 (refs 9, 10, 11). The aim of the current investigation was to determine if any other genetic information is cotransferred with the CTns. We demonstrate that the PaLoc can be transferred to CD37, which results in this non-toxigenic strain being converted to a toxin producer.

## Results

### The PaLoc transfers to non-toxigenic *C. difficile CD37*

To determine whether genetic elements were cotransferred in the absence of direct selection when transfer of CTn*1* was selected, we initially investigated nine CD37 transconjugants containing CTn*1*::*ermB* (an erythromycin-resistant derivative of CTn*1* (ref. 9)) by PCR and subsequent DNA sequencing (see Methods section for details). One of these strains had unexpectedly acquired the PaLoc. To determine whether the PaLoc could transfer independently of CTn*1*, strain 630Δ*erm tcdB::erm*(B) (Table 1)^4^ containing a genetically marked PaLoc was used as a donor with CD37 as the recipient. PaLoc-containing transconjugants were obtained at a frequency of 7.5 × 10^−9^ transconjugants per donor (s.d.=4.2 × 10^−9^), comparable to transfer frequencies previously reported for other CTns that are transferred between these strains^9^.

**Figure 1:**
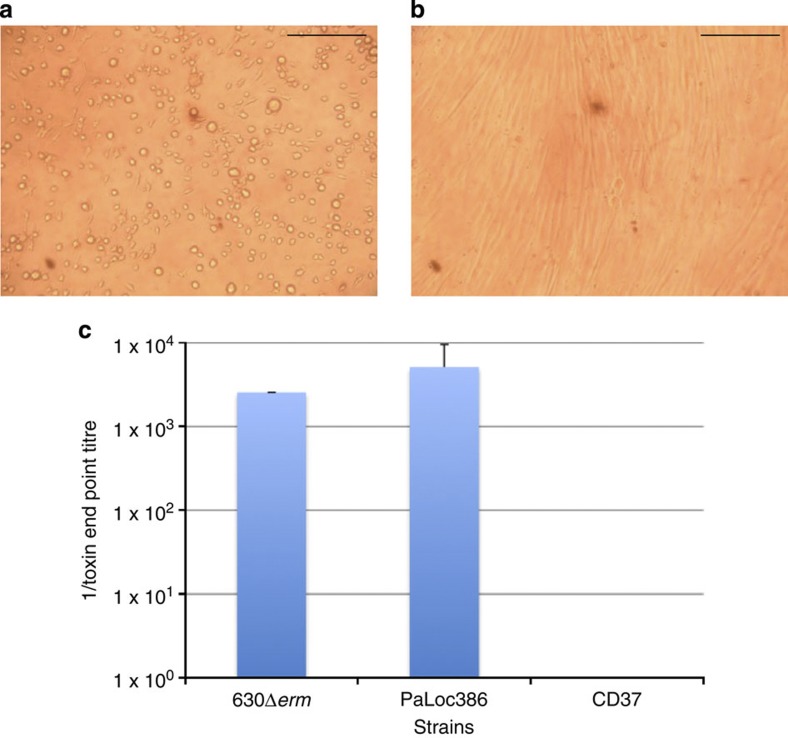
Cytotoxicity assay comparing toxin production. Toxin production by the donor, transconjugant and recipient strains were compared using a cytotoxicity assay. (**a**) HFF-1 cells incubated with the culture supernatant of transconjugant PaLoc386 show rounding of the cells. (**b**) The cytotoxic effect of PaLoc386 can be neutralized using antibodies against TcdB. Scale bar, 1 mm in **a** and **b**. (**c**) The toxin end point titre was determined for culture supernatants of donor strain 630 Derm, transconjugants PaLoc386 and the recipient CD37. The mean±s.d. is shown for three independent experiments.

It is important to determine whether the newly acquired toxin genes are capable of directing toxin production. To do this, one of the PaLoc-containing transconjugants that had acquired the wild-type PaLoc (that is, one of the transconjugants that contained CTn*1* and the PaLoc) was selected for further study and designated PaLoc386 (Table 1). This strain was subject to an *in vitro* cytotoxicity assay with HFF-1 cells. Filter-sterilized cell supernatants were incubated with a monolayer of HFF-1 cells for 24 h before the cytopathic effect (CPE) was determined (Fig. 1a). The end point toxin titre^4^ was determined for 630Δ*erm*, PaLoc386 and CD37 (Fig. 1c). PaLoc386 produced similar levels of toxin to the donor strain 630Δ*erm,* whereas supernatant from the recipient strain CD37 was negative for toxin production (Fig. 1c). Addition of a commercial TcdB antiserum (see Methods) abolished the CPE of both the 630Δ*erm* and PaLoc386 supernatants indicating that the two strains produced functional toxin B (Fig. 1c).

### The PaLoc is transferred on variable-sized DNA fragments

**Figure 2:**
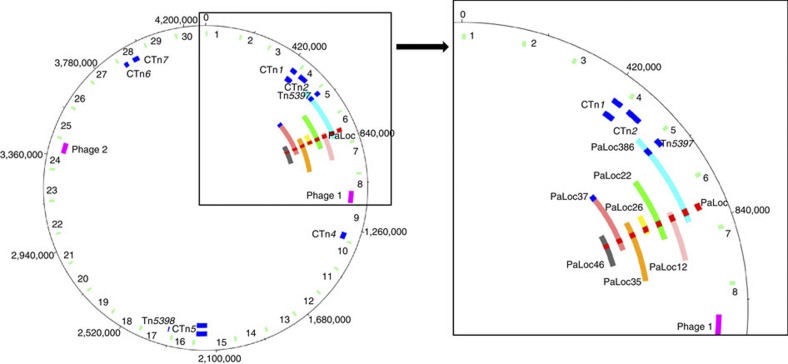
Schematic representation of the donor-specific DNA fragments present in the transconjugants. The circular chromosome of *C. difficile* strain 630 is shown. The boxed region on the left is magnified on the right. The green fragments in the outer ring represent the 10 kb fragments used to differentiate transconjugants from donors; see text for more details. The dark blue fragments in the second ring indicate the mobile genetic elements present in the donor; the red fragment in this ring represents the PaLoc. The donor-specific fragments that are present in the transconjugants are shown in the seven inner-most rings as coloured fragments with the number of each transconjugant indicated. The red fragments in these represent the transferred PaLoc. The dark blue fragments in the track of PaLoc386 depict CTn*1* and CTn*5* that are both present in this transconjugant.

To investigate the mechanism underlying the PaLoc transfer, whole-genome sequences were determined for seven transconjugants containing either the marked or wild-type PaLoc. The sequence of the PaLoc in strain PaLoc386 was identical to that of the donor. In the other transconjugants, the PaLoc contained the clostron insertion, but was otherwise identical to the PaLoc in 630Δ*erm*. To define the horizontally transferred region in the transconjugants, it was necessary to distinguish between donor and recipient genome. To do this, 30 regions of ~10 kb evenly spaced around the 630Δ*erm* genome were selected (shown schematically in Fig. 2 as green bars with the sequences given in SI1) and compared with the corresponding regions of the CD37 genome^12^. These regions were chosen as they did not contain any predicted mobile genetic elements such as CTns, IStrons or prophages. The presence of 10–150 single-nucleotide polymorphisms (SNPs) and indels in these 30 regions allowed discrimination between the donor and recipient DNA. Alignment of the genomic DNA sequence of the PaLoc-containing transconjugants in the 30 regions showed that in all cases, regions 1–3 and 8–30 were derived from the recipient. In the donor strains, 630Δ*erm* and 630Δ*erm tcdB::erm*(B), and in all of the transconjugants, the PaLoc is located between regions 6 and 7. In the transconjugants, the PaLoc has transferred on variable-sized DNA fragments that include either region 6 or 7 from the donor strain in 6 out of 7 transconjugants, and regions 5 and 6 from the donor in transconjugant PaLoc385. In transconjugant PaLoc26, the PaLoc was transferred from the donor on a relatively small DNA fragment (a maximum size of 67,644 bp) and both regions 6 and 7 in this transconjugant are derived from the recipient. In transconjugants, PaLoc386 and PaLoc37, Tn*5397* and the PaLoc have transferred on the same DNA fragment (Fig. 2). This contrasts with the other cotransfers where the CTn and the PaLoc transferred on separate DNA fragments. For example, in transconjugant PaLoc386, CTn*5* also transferred but independently of the PaLoc and has inserted near region 16 in the transconjugant (Fig. 2). In contrast to PaLoc transfer, in which the flanking DNA was also derived from the donor, there was no evidence for the transfer of flanking DNA in the case of CTn*5*. The remaining PaLoc-containing transconjugants shown in Fig. 2 did not contain CTn*2*, CTn*4*, CTn*5*, CTn*7* or Tn*5397*.

The approximate length of the transferred DNA in each of the transconjugants was determined by calculating the distance from the first SNP or indel upstream of the PaLoc to the last SNP or indel downstream of the PaLoc that was specific to the donor strain 630Δ*erm*. Because of the high level of sequence identity between CD37 and 630Δ*erm* in the regions flanking the PaLoc, this analysis could only determine the maximum and minimum size of the transferred fragment (Table 2, sequences included in SI2). The PaLoc was transferred on variable-sized DNA fragments ranging from at least 66,034 bp to a maximum of 272,977 bp (Table 2 and Fig. 2).

To exclude transformation as a mechanism of transfer, the mating experiments were repeated in the presence of DNase and no difference in the frequency of transfer was detected. It was also possible that the PaLoc could be transferred by transduction by either of the prophages that are present in strain 630Δ*erm* (Fig. 2). We think this is improbable given that the transferred DNA is much larger than the phage genomes and is unlikely therefore to be packaged within the phage heads. Nonetheless concentrated phage suspensions were made by mitomycin C induction of 630Δ*erm tcdB*::*erm*(B). We attempted to infect strain CD37 with these phage suspensions but did not obtain plaques, indicating that this strain is resistant to these phages. Strain CD843 was used to confirm the presence of infectious phages in the suspensions. Although plaques were produced with this host, no lincomycin-resistant transductants were obtained in this phage assay. These results indicate that PaLoc transfer is unlikely to occur via transduction.

### The PaLoc can transfer into other non-toxigenic strains

Recent work by Dingle *et al.*^13^, analysing the molecular epidemiology of a large population of *C. difficile* strains, has suggested that PaLoc transfer is occurring in wild populations. We tested two of the non-toxigenic isolates from this study (OX904 belonging to clade 4, PCR-ribotype 138 and OX2157 belonging to clade 1, PCR-ribotype 140) to determine whether they could act as recipients for the marked PaLoc in matings using 630Δ*erm tcdB::erm*(B) as donor. To do this, spontaneous rifampicin-resistant derivatives of these strains were first isolated (see Methods and Table 1). The PaLoc was transferred to OX2157rif at a frequency of 4.4 × 10^−8^ and to OX904rif at a frequency of 2.3 × 10^−9^, indicating that PaLoc transfer is not restricted to particular donor and recipient pairs.

## Discussion

This work demonstrates that the PaLoc of *C. difficile* 630Δ*erm* is capable of transfer by a conjugation-like mechanism to non-toxigenic strains. In the case of *C. difficile* CD37, we showed that this resulted in its conversion to a toxin producer. This finding has important clinical implications, as non-toxigenic strains have shown promise as a treatment for CDAD^14^. Clearly, if the PaLoc is capable of transfer to strains being used for treatment, then we urgently need to understand the conditions under which transfer of the PaLoc is selected. Non-toxigenic strains are found coexisting with toxigenic ones^13^, providing opportunity for the PaLoc to spread within the *C. difficile* populations. Also of concern is the finding that transfer of the PaLoc often, but not always, occurs with cotransfer of conjugative transposons encoding resistance to antibiotics, resulting in the cotransfer of virulence and antibiotic-resistance genes. This is illustrated in the transconjugants PaLoc386 and PaLoc37, in which Tn*5397* (encoding tetracycline resistance) and the PaLoc were transferred on the same DNA fragment from 630Δ*erm* to CD37. CTns and the PaLoc can also be transferred on separate DNA fragments as shown by transconjugant PaLoc386 in which CTn*5* and CTn*1* are transferred and integrated into their preferred genomic sites^9^ (between regions 15 and 16 for CTn*5*, and between regions 3 and 4 for CTn*1* (Fig. 2)) with no detectable transfer of flanking DNA. Presumably in this case, CTn*5* and CTn*1* transferred by excision to form a circular molecule that was nicked at the origin of transfer (*oriT*) and transferred as a distinct genetic element.

A striking observation is that although transfer of the PaLoc and the CTns occurs at a low frequency (between 10^−7^ and 10^−9^ transconjugants per donor), cotransfer of unselected genetic elements (including the PaLoc) is observed. A probable explanation for this is that when one or more of the CTns initiates a transfer event, other elements present in the same cell are activated by *trans-*acting regulatory molecules. This type of interaction has been observed between Tn*916*-like elements^15^. The first step in transfer by conjugative and mobilizable transposons is excision to form a circular molecule that is nicked at the *oriT*, and a single strand is transferred to a suitable recipient via a mating pore in a complex process analogous to type IV secretion^16^. However, the PaLoc is not contained within an obvious mobile element and is transferred on DNA fragments of variable size in different transconjugants. This type of transfer is reminiscent of that mediated by high-frequency recombination (Hfr) in which an *oriT* within the chromosome can mediate transfer of the chromosome^17^. In the recipient, the incoming chromosomal DNA is integrated into the chromosome by homologous recombination. In theory, the whole chromosome can be transferred, although this rarely happens due to disruption of the mating pair or nicking of the incoming DNA strand, so that markers proximal to the integrated *oriT* are transferred at a higher frequency than those located distally. There are examples of CTns mediating Hfr-type transfer, for example, in *Vibrio cholerae*^18^ and *Bacteroides* sp.^19^ In these cases, the CTns do not excise so that instead of just mobilizing themselves, the chromosome is also mobilized. Three transfer-proficient CTns in the *C. difficile* genome, CTn*1*, CTn*2* and Tn*5397* are close to the PaLoc (Fig. 2) and are candidates for mediation of Hfr transfer. The regions flanking the PaLoc in 630Δ*erm* are homologous to chromosomal DNA of the non-toxigenic strain, CD37, and, consequently, the incoming DNA from the donor could recombine with the recipient chromosome resulting in integration of this region. Whole-genome sequence comparison has indicated that large blocks of DNA have transferred between *C. difficile* strains in the past, indicating that this form of transfer is an important driver of *C. difficile* genome evolution^20^.

A recent study of diversity in the *C. difficile* S-layer cassette suggests that this cassette is undergoing horizontal gene transfer across genotypes^21^. The recombination events for the cassette shown in this study were significantly smaller (12–35 kb) than those reported here for the PaLoc, and the recombining loci are located ~2 Mb apart on the chromosome. Although transfer of the S-layer cassette has not been demonstrated, it is possible that this region can transfer by a mechanism similar to that of the PaLoc, a hypothesis that requires investigation.

## Methods

### Strains and growth conditions

All strains used in this study are listed in SI 3. *C. difficile* strains were grown in brain–heart infusion (BHI) broth or on BHI agar (Oxoid Ltd, Basingstoke, UK) supplemented with 5% defibrinated horse blood (E & O laboratories, Bonnybridge, UK). For the cytotoxicity assay, *C. difficile* was grown in tryptone, yeast (TY) broth consisting of 3% Bacto Tryptose (BD Biosciences, Oxford, UK), 2% yeast extract (Oxoid Ltd) and 0.1% thioglycolate (Sigma-Aldrich Company Ltd, UK) at pH 7.4. *C. difficile* strains were grown at 37 °C under anaerobic conditions (80% N_2_, 10% H_2_ and 10% CO_2_).

### Molecular biology techniques

DNA was isolated using the Gentra Puregene Yeast/Bact. Kit (Qiagen, UK). PCR amplification was carried out using the NEB Taq polymerase kit (New England Biolabs, UK) according to the manufacturer’s instructions.

### Filter-matings

Donor and recipient strains were grown in BHI broth to an OD_600_ of ~0.5–0.6. Cells were washed, pellets were taken up into BHI broth and donor and recipient cultures were mixed. The cell mixture was spread onto 0.45 μm pore size cellulose nitrate filters (Sartorius, Epsom, UK) on BHI agar plates. In some cases DNase was added to the mating plates at a concentration of 50 μg ml^−1^. After 24 h, the filters were placed in 25 ml tubes containing 1 ml BHI broth and were vortexed vigorously. The cell mixture was spread onto selective plates to determine donor, recipient and transconjugant cell numbers on the filters.

Putative transconjugants were assessed by PCR for the presence of the *erm*(B) selection marker using primers ErmRAM-F (5′-ACGCGTTATATTGATAAAAATAATAATAGTGGG-3′) and ErmRAM-R (5′-ACGCGTGCGACTCATAGAATTATTTCCTCCCG-3′)^22^. The presence of the PaLoc was determined by PCR using primers tcdA-F (5′-CATCTATAAGTTCTCATATTCCTTC-3′) and tcdA-R (5′-CCAGAAACTTATATTGTCC-3′). The absence of the CDT locus was determined using primers cdtA-F (5′-(C/T)AATACTACTTACAAGGCTCC-3′) and cdtA-R (5′-TTTCGTTTTGATTTT(C/T)TGTTC-3′). Spontaneous rifampicin-resistant mutants were isolated by plating 10^10^ bacteria on agar plates containing 25 μg ml^−1^ rifampicin. As the complete genome sequence of strains OX904 and OX2157 is not available, transconjugants were differentiated from spontaneous rifampicin-resistant mutants of the donor by PCR and sequencing of genes *cwp66* (primers 5′AAACCWAHARYYAATGATACAG and 5′RTWGWTYTTCCCCATCKWGAAAC) and gene CD25780 (primers 5′GATGATATAGACTTTACATCAGAAG and 5′CAGCCGCAATTACTTTTAATC) encoding a predicted serine threonine protein kinase.

### Cytotoxicity assay

HFF-1 cells were grown in Dulbecco’s Modified Eagle Medium (DMEM) (Life technologies) supplemented with 10% heat-inactivated fetal calf serum, 2 mM glutamine, 100 units ml^−1^ penicillin and 100 μg ml^−1^ streptomycin at 37 °C in 5% CO_2_. Cells were subcultured by washing twice with sterile PBS and incubating with 0.1% trypsin for 5 min. For the cytotoxicity assay, the cells were seeded at 10^4^ cells per well in 96-well plates and incubated for 24 h in 5% CO_2_.

Overnight *C. difficile* cultures were diluted 1:10 into tryptone, yeast (TY) broth and incubated without shaking for 48 h. The cultures were centrifuged for 10 min at 4,500 *g* and the supernatant was filter-sterilized. A dilution series of the supernatant was prepared in PBS. Each dilution was added to confluent HFF-1 cells in duplicate. After 24 h, the cells were assessed for CPE using an inverted microscope. The toxin end point titre was recorded as the lowest dilution in the series where the cells were indistinguishable from the negative control. *C. difficile* antitoxin (*C. difficile* test kit, T5003Techlab Inc, Blacksburg, VA, USA) was added to one of the duplicate wells to determine whether the CPE was neutralized.

### Genomic sequencing

Genomic sequencing was performed by UCL Genomics as part of a 72-bp paired end run, performed on an Illumina GAII-X. Genome assembly was performed using the Velvet software suite^23^ and reads were mapped against strain 630 (AM180355) using xBase^24^. The transconjugant sequences were compared with the sequence of strain 630 (AM180355) and the shotgun sequence of strain CD37 (AHJJ01000000) using the Artemis Comparison Tool^25^. SNP analysis was performed by alignment using eBioX version 1.51. Sequence of the chromosomal DNA used to compare the donor- and recipient-specific DNA in the transconjugants is provided in Supplementary Data 1. Sequence at the junctions between the chromosome and the donor-specific fragment is provided in Supplementary Data 2. The ends on either side of the transferred fragment are compared with the donor and recipient sequences.

## Author contributions

M.S.M.B., P.M., E.A. and A.P.R. designed the experiments and wrote the manuscript. M.S.M.B., R.W. and H.H. carried out the experiments.

## Additional information

**How to cite this article:** Brouwer, M. S. M. *et al.* Horizontal gene transfer converts non-toxigenic *Clostridium difficile* strains into toxin producers. *Nat. Commun.* 4:2601 doi: 10.1038/ncomms3601 (2013).

## Supplementary Material

Supplementary Dataset 1 Sequence of the chromosomal DNA used to compare the donor- and recipient-specific DNA in the transconjugants.

Supplementary Dataset 2 Sequence at the junctions between the chromosome and the donor specific fragment. The ends on either side of the transferred fragment are compared to the donor and recipient sequences.

## Figures and Tables

**Table 1:** Bacterial strains used in this study.

**Strain (Ribotype)**	**Properties**	**Reference or source**
630 (012)	Tc^R^ Erm^R^ Rif^S^	26
630Δ*erm* (012)	Tc^R^ Erm^S^ Rif^S^	6
CD37 (009)	Tc^S^ Erm^S^ Rif^R^ non-toxigenic	27
CD843	Positive control for ϕC630-1 and ϕC630-2	28
630Δ*erm* CTn*1* CD0386::*erm*(B) (012)	ClosTron mutant, donor of CTn*1*, *erm*(B) present in CD0386 within CTn*1*	9
630Δ*erm* tcdB::*erm*(B) (012)	ClosTron mutant, contains *erm*(B) inserted within tcdB	4
OX904 (138)	Non-toxigenic recent clinical isolate	13
OX2157 (140)	Non-toxigenic recent clinical isolate	13
OX904rif (138)	Rifampicin-resistant derivative of OX904	This study
OX2157rif (140)	Rifampicin-resistant derivative of OX2157	This study
PaLoc386	CD37 transconjugant containing the 630Δ*erm* wild-type PaLoc	This study
PaLoc12	CD37 transconjugant containing the ClosTron *tcdB*::*erm*(B) PaLoc	This study
PaLoc22	CD37 transconjugant containing the ClosTron *tcdB*::*erm*(B) PaLoc	This study
PaLoc26	CD37 transconjugant containing the ClosTron *tcdB*::*erm*(B) PaLoc	This study
PaLoc35	CD37 transconjugant containing the ClosTron *tcdB*::*erm*(B) PaLoc	This study
PaLoc37	CD37 transconjugant containing the ClosTron *tcdB*::*erm*(B) PaLoc	This study
PaLoc46	CD37 transconjugant containing the ClosTron *tcdB*::*erm*(B) PaLoc	This study

Erm, erythromycin; Rif, rifampicin; R, resistant; S, susceptible; Tc, tetracycline.

**Table 2:** Size of the DNA fragments inserted in the recipient strain.

**Isolate**	**Minimum size of DNA fragment transferred**	**Maximum size of DNA fragment transferred**
PaLoc386	271,992 bp	272,977 bp
PaLoc12	151,692 bp	155,024 bp
PaLoc22	157,688 bp	170,386 bp
PaLoc26	66,034 bp	67,644 bp
PaLoc35	224,973 bp	228,497 bp
PaLoc37	250,027 bp	252,009 bp
PaLoc46	141,658 bp	145,290 bp
